# Analysis of Spatial Distribution and Prognostic Value of Different Pan Cytokeratin Immunostaining Intensities in Breast Tumor Tissue Sections

**DOI:** 10.3390/ijms21124434

**Published:** 2020-06-22

**Authors:** Velicko Vranes, Tijana Vujasinović, Nemanja Rajković, Ksenija Kanjer, Nebojša T. Milošević, Marko Radulovic

**Affiliations:** 1 Department of Basic and Environmental Science, Instituto Tecnológico de Santo Domingo (INTEC), Santo Domingo 10602, Dominican Republic; velicko.vranes@intec.edu.do; 2 Department of Experimental Oncology, Institute for Oncology and Radiology, 11000 Belgrade, Serbia; tijana.vujasinovic@bio-techne.com (T.V.); kanjer@ncrc.ac.rs (K.K.); 3 Department of Biophysics, School of Medicine, University of Belgrade, 11000 Belgrade, Serbia; nemanja.rajkovic@med.bg.ac.rs (N.R.); nebojsa.milosevic@med.bg.ac.rs (N.T.M.)

**Keywords:** breast cancer, prognosis, metastasis, image analysis, pan cytokeratin, histopathology, epithelial, immunostaining, intensity slicing

## Abstract

Cancer risk prognosis could improve patient survival through early personalized treatment decisions. This is the first systematic analysis of the spatial and prognostic distribution of different pan cytokeratin immunostaining intensities in breast tumors. The prognostic model included 102 breast carcinoma patients, with distant metastasis occurrence as the endpoint. We segmented the full intensity range (0–255) of pan cytokeratin digitized immunostaining into seven discrete narrow grey level ranges: 0–130, 130–160, 160–180, 180–200, 200–220, 220–240, and 240–255. These images were subsequently examined by 33 major (GLCM), fractal and first-order statistics computational analysis features. Interestingly, while moderate intensities were strongly associated with metastasis outcome, high intensities of pan cytokeratin immunostaining provided no prognostic value even after an exhaustive computational analysis. The intense pan cytokeratin immunostaining was also relatively rare, suggesting the low differentiation state of epithelial cells. The observed variability in immunostaining intensities highlighted the intratumoral heterogeneity of the malignant cells and its association with a poor disease outcome. The prognostic importance of the moderate intensity range established by complex computational morphology analyses was supported by simple measurements of its immunostaining area which was associated with favorable disease outcome. This study reveals intratumoral heterogeneity of the pan cytokeratin immunostaining together with the prognostic evaluation and spatial distribution of its discrete intensities.

## 1. Introduction

As a primary breast tumor is not life-threatening, the outcome of breast cancer depends on metastasis occurrence, which is the main cause of death [1]. Patients are thus treated with cytotoxic therapy to eliminate distant micrometastases. However, most breast cancer patients do not incur metastasis even without cytotoxic chemotherapy and are thus unnecessarily exposed to toxic side effects of chemotherapy treatment [2,3]. This issue could be resolved by the precision medicine, with less intense treatments for those at low risk and more intense ones for those at a reliably established high metastasis risk. Improvement of the breast cancer patient survival rate through such individualized adjustment of chemotherapy is still not possible only because the risk of metastasis occurrence cannot be reliably prognosticated.

Traditional prognostic indicators include histologic grade which exploits histomorphological features such as tubule formation, nuclear pleomorphism, cell growth structures, and mitotic cells [4]. TNM staging considers clinicopathological features such as tumor size, lymph node spread, and metastasis. The molecular prognostic indicators include steroid receptor status, HER2 amplification [5], and gene signatures such as MammaPrint and Oncotype DX [6,7]. Intrinsic or molecular classification of breast cancer into luminal A, luminal B, basal, normal-like, triple-negative/basal-like, and HER2-enriched also presents prognostic value [8]. However, the current prognosis of metastasis risk is still not sufficiently reliable. Even the most advanced gene signature tools deliver an accuracy of only 65% and an area under the ROC curve (AUC) of 0.69 [9].

The pressing need for the improvement of breast cancer outcome prognosis stimulates intensive research aimed at quantification of intertumoral heterogeneity as a means for classification of tumors according to their metastatic potential. Breast cancer inter- and even intratumoral heterogeneity has been demonstrated in genomic, histologic, and radiologic analyses.

We hypothesized that the current prognostic biomarkers do not provide sufficient reliability, because they do not fully exploit tumor heterogeneity. Histologic grade analyses concern growth patterns of malignant cells which reflect the sum of molecular interactions within a tumor and thus present a rich source of tumor heterogeneity information. However, we intended to extend the reach of histologic grade by using the computational analysis as an emerging approach designed to exploit information that cannot be quantified by microscopic inspection, such as spatial distribution, texture, shape and complexity [10,11,12,13,14]. Its advantages also include high speed and cost-efficiency. Unlike the traditional practice of visual interpretation of medical images, we treat images as mineable data by extracting quantitative features. This approach gains in importance with improvements in computational power and the availability of the whole slide digital scanners [15].

Epithelial tissue is the most common site of malignant transformation, accounting for as many as 90 per cent of all human cancers. The prevalence of epithelial cancers may be due to very high cell division frequency of epithelial cells. Both normal and malignant epithelial cells can be stained with AE1 and AE3 monoclonal antibodies that are well-defined and have high specificity for keratin fibers [16]. Malignant epithelial cells are usually organized in tumor nests of variable sizes and shapes scattered throughout stroma which contains mostly fibroblasts, some immune cells, and few epithelial buds [17]. We have previously found that low pan cytokeratin staining intensity is associated with a poor breast cancer outcome [18].

Intrigued by our previous finding that low pan cytokeratin staining intensity is associated with a poor outcome [18], we set out to perform a detailed computational investigation of the spatial distribution and prognostic value of the discrete pan cytokeratin staining intensities.

## 2. Results

### 2.1. The Prognostic Model for Distant Metastasis Risk

The major advantage of the used patient group was that treatments were only local in terms of surgery and radiation, without systemic hormonal or cytotoxic drugs which could interfere with metastasis occurrence. This treatment protocol was in line with the recommendations effective in the year 1993 for breast carcinoma patients classified at a lower risk based on early breast cancer with tumors of smaller pathological tumor size pT1 and pT2, histologic grade 1 and 2. With both TNM and histologic grade being ineffective in this early breast cancer patient group without lymph node involvement or metastasis (N0M0), only tumor size showed a prognostically significant association by an AUC of 0.65 (95% CI = 0.51–0.78; *p* = 0.04). For the distribution of clinicopathological parameters in this group of patients, including age, tumor size, histologic grade, estrogen receptor (ER), progesterone receptor (PR), HER2 status and metastasis location, please refer to our previous report [17].

### 2.2. Spatial Distribution of the Pan Cytokeratin Immunostaining Intensities

**Figure 1:**
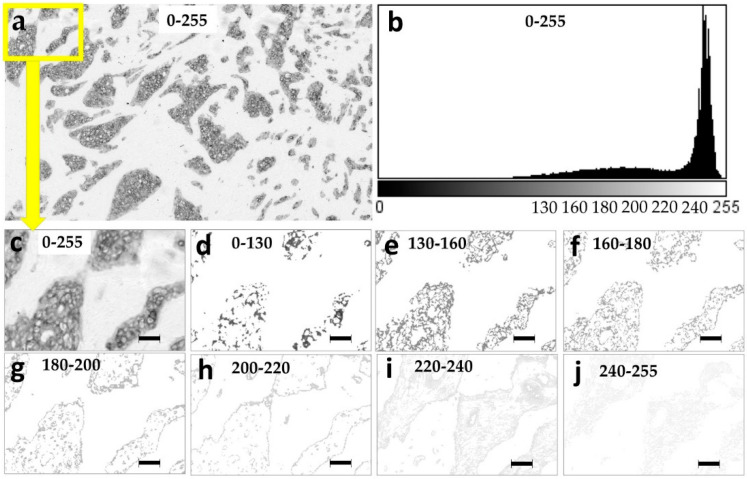
Grey level slicing of the exemplary breast tumor tissue section stained with pan cytokeratin. (**a**) Original image with the full 0–255 pixel intensity range, (**b**) intensity histogram of the original 0–255 spectrum with pixel intensity on the x axis versus a number of pixels on the y axis. Magnified inserts of the original image show: (**c**) the original grey level range of 0–255, (**d**) 0–130 grey level range, (**e**) 130–160, (**f**) 160–180, (**g**) 180–200, (**h**) 200–220, (**i**) 220–240, and (**j**) 240–255 grey level ranges. Magnification in (**c**–**j**): ×200. Pixel size: 1.4 µm. Bar 50 μm, indicated in images 2c–j.

**Figure 2:**
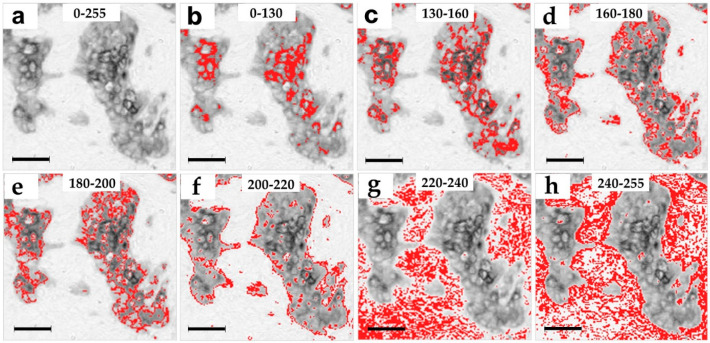
Spatial distribution of the pan cytokeratin staining intensities. (**a**) Magnification of the exemplary original image of pan cytokeratin staining with the full 0–255 pixel intensity range, (**b**) the original image overlaid with red pixels indicating the staining patterns in the highest intensity range of 0–130 and the moderate intensity ranges: (**c**) 130–160, (**d**) 160–180, (**e**) 180–200, and the low intensity ranges of (**f**) 200–220, (**g**) 220–240, and (**h**) 240–255. Magnification: ×320. Pixel size: 1.8 µm. Bar 50 μm, indicated in images (**a–h**).

**Figure 3:**
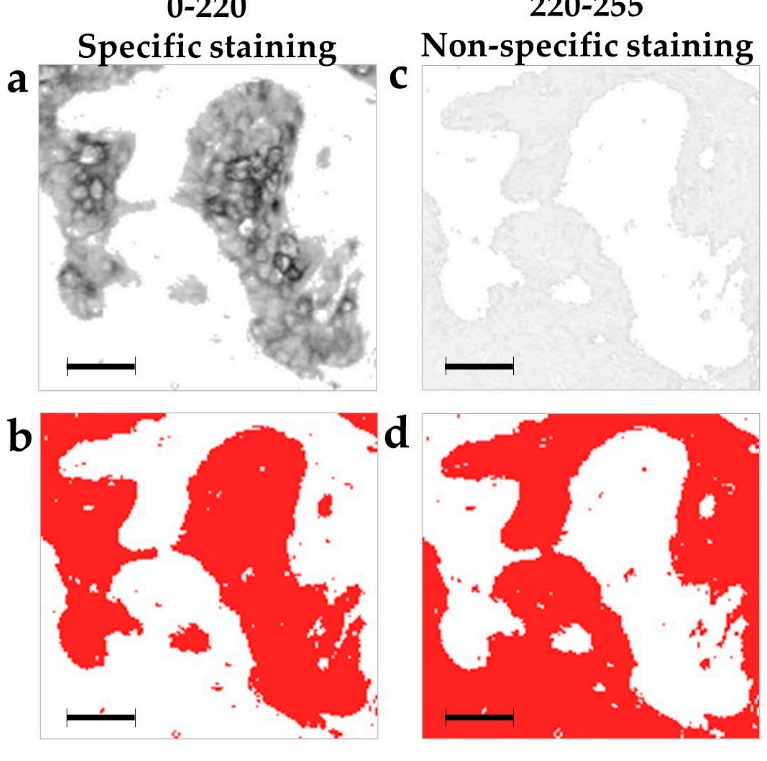
Specific and non-specific pan cytokeratin staining. The pan cytokeratin staining intensity cutoff at the 220 grey-level separates the immunostaining of the epithelial and stromal tumor areas. (**a**) Exemplary pan cytokeratin immunostaining within the specific 0–220 pixel intensity range; (**b**) the binary mask of the previous image accentuates the pattern of specific staining; (**c**) non-specific staining in the pixel intensity range of 220–255; (**d**) the binary mask of the previous image accentuates the non-specific staining pattern. Magnification: ×320. Pixel size: 1.8 µm. Bar 50 μm, indicated in images (**a–d**).

Grayscale images are composed of pixels containing only light intensity information where zero is taken to be black and 255 is taken to be white. We performed the pixel intensity level slicing in order to achieve a separate visualization of different pan cytokeratin immunostaining intensities. Each original image (Figure 1a,b) was thereby segmented into seven separate images with a single narrow pixel intensity level range: 0–130, 130–160, 160–180, 180–200, 200–220, 220–240, and 240–255 (Figure 1d–j). Most pixels were distributed in the moderate and weak pixel intensity ranges from 150–250 as seen in the intensity histogram of an exemplary image (Figure 1b). Based on such distribution, the 0–130 and 130–160 ranges were wider, because these contained a smaller fraction of pixels (Figure 1b). The average distribution of pixel-intensities was 6% in the 0–130 intensity range, 9% in the 130–160 range, 10% in the 160–180 range, 8% in the 180–200 range, 8% in the 200–220 range, 29% in the 220–240 range, and 30% in the 240–255 range. Pan cytokeratin clearly stains the patches of malignant epithelial cells (Figure 1a,c). Pixel intensity slicing revealed that high and moderate intensities in the 0–200 range were distributed within these patches (Figure 1d–g), the weaker 200–220 range mostly stained the patch borders (Figure 1h), while the weakest grey levels of 220–255 stained the stroma (Figure 1i,j). The spatial pan cytokeratin distribution is more easily observed in images with overlapped original greyscale immunostaining (Figure 2a) and the narrow grey level ranges marked by red pixels (Figure 2b–h). This figure clearly indicates that pixel intensity ranges from 0 to 220 cover the area within tumor nests, which is compatible with the distribution of epithelial cells. We thus set a cutoff between the specific and non-specific staining at the 220 grey level. The entire specific staining pattern within the range of intensities from 0 to 220 is presented in Figure 3a with its binary image mask in Figure 3c for comparison with the non-specific staining in the weaker intensity range from 220 to 255 (Figure 3b) and its binary mask in Figure 3d.

### 2.3. Prognostic Evaluation of the Distinct Pan Cytokeratin Staining Intensities

Our strategy was to evaluate the distribution of prognostic information among the seven narrow grey level ranges of the pan cytokeratin immunostaining in tumor tissue sections. All images were analyzed by the five first-order statistics, five (GLCM), and 23 monofractal features. Prognostic evaluation of the calculated features was achieved by the receiver operating characteristic (ROC) analysis with the distant metastasis occurrence as the endpoint. Table 1 presents features which reached the prognostic significance in at least one grey level intensity range. AUC = 0.5 represents chance discrimination, while perfect discrimination equals 0.0 or 1.0.

Pixel grey level slicing introduced a large number of white pixels as a substitute for the deleted intensities outside of the highlighted intensity ranges (Figure 1d–j). It is important to note that GLCM and fractal analysis took into account all of the newly introduced white pixels. However, the first-order statistics could be calculated strictly within the designated grey level ranges without consideration of the white 255 grey level pixels.

Table 1 shows that all three types of image analysis algorithms provided prognostic significance in the original images and the narrow grey level ranges (Table 1). The poorest prognostic performance was provided by the 0–130 range where none of the features reached prognostic significance. Two features provided prognostic significance in the 200–220 range, while other ranges provided 3–9 significant features (Table 1). The prognostic performance of several features was improved in the narrow greyscale ranges in comparison to the original images. These were typically the features which did not show prognostic significance in the original images, such as contrast, correlation, D_B_, and SE for D_B_ (Table 1).

Interestingly, the prognostic improvement of the features that were already significant in the original images was only noted for the first-order statistical features: mean and kurtosis (Table 1). Kurtosis is a feature measuring the distance of outliers in the pixel intensity distribution tails.

To identify the prognostically optimal pixel intensity range, we calculated the average AUC value for each range (Table 2). Additionally, we calculated the sum of all prognostically significant AUC improvements in the narrow ranges in comparison to the original images as absolute distances of significant AUC values from the null hypothesis value of 0.5 (Table 2). We found that the three middle range intervals of pixel intensities (between 130 and 200) provided the best prognostic performance with AUCs ranging between 0.64–0.67 (Table 2). This prognostic performance was in line with the average AUC of 0.66 calculated for the original greyscale images (Table 2). The average improvement of prognostic performance was also highest in the ranges spanning from 130 to 200, with the 160–180 intensity interval providing the best prognostic improvement.

### 2.4. Prognostic Independence of the Features Calculated in Narrow Ranges of Pan Cytokeratin Immunostaining Intensities

Multivariate binary logistic regression analysis (Table 3) included the demographic (age), clinicopathological (ER and tumor size), and the 38 image analysis features calculated in the original images and narrow ranges satisfying the selection entry criterion of *p* ≤ 0.05 obtained by the ROC analysis (Table 1 and [17]). We used continuous values for this test to avoid the bias introduced by categorization. Table 3 presents only the remaining variables, indicating the non-redundant prognostic value for the two features obtained in narrow pan cytokeratin immunostaining intensity ranges: D_B_ and kurtosis (Table 3). The prognostic independence of D_B_ supported the prognostic importance of the 160–180 intensity range indicated in Table 2.

## 3. Discussion

Immunostaining of keratin reveals the shapes, sizes, and distribution of malignant cell patches in cancers caused by the neoplastic transformation of epithelial cells and thus presents a paramount prognostic potential. While the cytokeratin staining of malignant nests [16] and their prognostic value in breast tumors [17,18,19,20,21] have been established, this is the first systemic analysis of the pan cytokeratin immunostaining based on its intensity. We revealed the spatial distribution and prognostic value for each of the produced seven narrow ranges of pan cytokeratin immunostaining intensities.

Our previous report indicated that the low intensity of pan cytokeratin immunostaining is associated with a higher metastasis risk [18]. The separation of immunostaining intensities allowed us to extend the previous study by providing a detailed insight into the prognostic performance of pan cytokeratin. We showed that narrow ranges of pan cytokeratin immunostaining intensities still provided sufficient tumor heterogeneity information to support the prognostically significant performance of image analysis features. The most astonishing surprises occurred at high and low intensity extremes. We showed that the most intense immunostaining comprising the darker half (0–130) of the entire greyscale spectrum (0–255) did not provide significant prognostic performance by any of the 33 calculated GLCM, fractal, and first-order statistical features. This result suggested the prognostic irrelevance of the differentiated epithelial cells with high cytokeratin expression [22]. All other intensity ranges offered 2–9 prognostically significant features, whereby moderate pixel intensity ranges (130–180) provided the best performance. Fractal and GLCM features indicated that low complexity (by D_B_), low entropy (by entropy), low contrast (by contrast) and high heterogeneity (by Λ and SE for D_B_) in this range prognosticated a poor outcome. The SE for D_B_ feature is a readout of image heterogeneity as a deviation of fractal dimension values calculated at different fractal grid positions within an image. In addition to such complex fractal and GLCM features, the prognostic importance of the 130–180 range was also supported by the simpler and very obvious feature of immunostaining area. It was prognostically significant only within this range with the low staining area prognosticating a poor outcome. This result was surprising, but in line with the previous reports showing that a smaller area of malignant cells in tumors indicates a poor outcome in breast [23] and other types of cancer [24,25]. It might be explained by the indirect effect of the large malignant areas in a reduction of stroma which promotes the development of pro-tumor immune infiltrates [26]. It was also reported that a high tumor/stroma ratio is associated with low peritumoral inflammatory infiltrates in patients with colorectal cancer [27].

We assume that the observed wide variation in pan cytokeratin staining intensities among epithelial cells reflects the differences in cytokeratin content and expression of different subtypes. Such variability in pan cytokeratin staining was noticeable not only within individual tumor nests, but even between neighboring cells. This result highlights the intratumoral heterogeneity of the malignant clones and also indicates that higher heterogeneity prognosticated a poor disease outcome. This conclusion was in line with the previous report that intratumoral heterogeneity based on HER2 expression is associated with a poor outcome [28]. The high variation of pan cytokeratin staining intensity did not necessarily indicate the different genetic clonality of neighboring cells, but was probably mostly due to epigenetic alterations [29]. The observed intratumoral phenotypic heterogeneity may be the basis for the good prognostic performance of the heterogeneity measures Λ and SD for D_B_ [28]. Single-cell heterogeneity has been previously investigated in breast cancer [30], but the current study shows for the first time that even broad pan cytokeratin immunostaining is a good measure for intratumoral heterogeneity. Evaluation of intratumoral heterogeneity is important because of its prognostic relevance and for presenting a major obstacle in effective cancer treatment [29].

Using grey level slicing, we could visualize the structures marked by different intensities of pan cytokeratin immunostaining. The most intense staining in the 0–130 range marked only a few cells within malignant nests, while weaker staining up to the level 200 was scattered throughout the malignant cell patches, also called tumor nests. It should be pointed out that higher grey values represent lighter pixels and thus lower intensity of staining. Still lighter shades of grey in the range of 200–220 mostly stained the borders of tumor nests, while the lightest 220–255 staining was found only in the stroma. As cytokeratins are expressed in epithelial cells which populate tumor nests and only sporadically found in the stroma [17,31], we assumed that the intensity level 220 was the precise cutoff between the specific 0–220 pan cytokeratin staining that was limited to tumor nests and the non-specific 220–255 staining that was almost entirely restricted to the stroma. Interestingly, while image analysis features exerted good prognostic performance within narrow grey level ranges, their pooling into a single wide 0–220 range could not improve the prognostic performance. It is thus evident that the original images which included specific as well as non-specific staining provided better prognostic performance in comparison to the range(s) containing only specific pan cytokeratin staining. This finding was in agreement with the unexpected prognostic value provided by the lowest intensity and non-specific immunostaining for features such as area, mean, kurtosis, Angular Second Moment (ASM), entropy and correlation. Taken together, these findings point to the prognostic value of stroma. The exactly inverse prognostic association of the specific and non-specific staining areas could be explained by their mutual dependence and high negative correlation. Interestingly, GLCM features ASM and entropy also showed an inverse association with the outcome in specific and non-specific staining intensity ranges, while the correlation feature consistently prognosticated a high metastasis risk. It was also evident that only fractal features provided prognostic significance only within the specific range of pan cytokeratin staining.

With its share in the specific immunostaining of only 15%, the intense dark staining was relatively rare, while lighter pixels were very abundant. This pixel intensity distribution was the reason for the width of 130 grey levels at the darker side of the image histogram (0–130), whereas lighter ranges were only 15–30 grey level-wide. The rarity of intense staining was not due to the low number of epithelial cells, as specific staining covered on average 42.8% of the image area. It was previously shown that mature epithelial cells stain more intensely for cytokeratins in comparison to less differentiated cells [22,32]. Therefore, the rarity of intense pan cytokeratin immunostaining could be explained by the overwhelmingly low differentiation state of epithelial cells.

The size of the patient group is a limitation of this study, although it exceeded the requirement estimated using the prospective sample size analysis. Additional validation with studies in an extended patient group and external groups is needed to examine whether any of the features obtained in this study in the narrow pixel intensity ranges could be combined with other prognostic parameters to develop the models that may offer a major improvement of prognostic accuracy. Another limitation was that the pan cytokeratin AE1/AE3 antibody cocktail immunostains both normal and malignant breast epithelial cells. We largely overcame this limitation by selecting the predominantly malignant tumor areas based on morphological criteria. Therefore, the pan cytokeratin staining in the current study indicated the growth patterns of malignant cells. However, the selection step by an expert pathologist included subjectivity into this computational analysis technique which is otherwise entirely objective. The advantages of this study included the untreated patient group with no systemic treatments which could interfere with metastasis outcome. Furthermore, this early breast cancer patient group with negative lymph node spread and distant metastasis and small tumor size lacks the TNM staging as the major prognostic factor. Even the histologic grade is uniformly low and therefore of limited prognostic use. Therefore, such a patient group is in particular need of novel prognostic markers.

## 4. Materials and Methods

This report was written to include all relevant experimental details according to the recommendations for tumor marker prognostic studies [33].

### 4.1. Ethical Approval Statement

The study was approved by the Ethics Committee of the Institute for Oncology and Radiology (#2794-01; 14. July 2016) and conforms with The Code of Ethics of the World Medical Association (Declaration of Helsinki) printed in the British Medical Journal (July 18, 1964) and its 7^th^ revision in 2013.

### 4.2. Patient Group

The patient group consisted of 102 female Caucasian women treated in the same year (1993) at the Institute of Oncology and Radiology of Serbia. We obtained the patient data in a deidentified form without identifiers that could enable reidentification (Safe harbor methodology of the Health Insurance Portability and Accountability Act of 2012). Estrogen receptor (ER) positivity was 69%, and 24% were positive for the progesterone receptor. The dextran-coated charcoal assay was used for measurements of estrogen and progesterone receptors [34]. The median age at diagnosis was 57 years (range: 37–80). The follow-up time for patients without metastasis ranged from 77 to 165 months with a median of 147 months (reverse Kaplan–Meier method). The time to metastasis ranged between 16 and 155 months with a median of 61 months. Amplification of the HER2 gene was detected in 22 patients. For a detailed description of this patient group in regard to HER2 and other clinicopathological parameters, please refer to our previous report [17].

The prospective sample size calculation was based on a pilot study including 40 patients and required 80 patients with 16 positive cases for alpha = 0.05, beta = 0.20, and AUC effect size of 0.67/0.33 (MedCalc Software, Ostend, Belgium). The obtained average significant absolute value of AUC was 0.68 with a sample size of 102 patients, of which 20 cases were metastasis-positive.

### 4.3. Study Workflow

Representative histopathology sections from 102 patients were selected by an expert pathologist and immunostained by a pan cytokeratin antibody to label epithelial cell clusters. The stained sections were digitized by a slide scanner and five representative areas were selected by an expert pathologist. The images were subjected to greyscale slicing in order to highlight seven non-overlapping pixel intensity ranges. Fractal and GLCM image analysis algorithms were subsequently used for the extraction of quantitative features followed by statistical analysis and validation of their prognostic performance.

### 4.4. Preparation of Tumor Tissue Sections

Tumor tissue was obtained during surgical removal. Tissue was formalin-fixed, paraffin-embedded, and cut to produce 4 µm whole sections.

### 4.5. Selection of Tissue Sections

To achieve maximal reproducibility and validity, the pathologist (K.K.) selected one whole tissue section per patient that best represented each individual tumor.

### 4.6. Immunostaining

Heat-mediated antigen retrieval was made in a water bath set to 95 °C for 40 min in a EDTA pH 8 buffer. Endogenous peroxidase was quenched with 3% H_2_O_2_ in methanol for 30 min and 5% goat serum was used for preincubation. Immunostaining was performed with pan cytokeratin primary antibody clones mAE1 and AE3 (Dako, Glostrup, Denmark, #M3515) and the CD8 monoclonal rabbit antibody (ThermoFisher Scientific, Waltham, MA, USA; #RM-9116-S1) as previously explained in detail [17]. The AE1/AE3 antibody cocktail stains epithelial cells by detecting 13 cytokeratins: 1–8, 10, 14–16, and 19. Counterstain was not performed in order to highlight only epithelial cells.

### 4.7. Image Acquisition

We acquired color images using a Hamamatsu NanoZoomer-XRC12000 high-resolution digital slide scanner (Hamamatsu City, Japan).

### 4.8. Image Selection

From the large area of each whole tissue section, the pathologist (K.K.) selected five representative areas containing the growth patterns characteristic for each individual tumor with the highest content of pan cytokeratin-stained malignant cells and without artefacts. Pan cytokeratin-stained cell arrangements were identified as normal or malignant according to their morphology.

### 4.9. Stain Decomposition

Blue (pan cytokeratin) and brown (CD8) channels were decomposed as previously described in detail by Li and Plataniotis [35]. All downstream image analysis in this study was performed in the blue pan cytokeratin channel.

### 4.10. Creation of the Images with Narrow Pixel Intensity Ranges

Grey level slicing was used to segment images into seven discrete narrow grey level ranges. Each range was made by conversion of all grey levels outside of the narrow range into white 255 pixels. For instance, a grey level range of 160 to 180 was highlighted in two steps by the following commands: run (“Macro...”, “code = [ if (v > = 0 && v< = 160) v = 255]”) and run (“Macro...”, “code = [ if (v> = 180 && v< = 255) v = 255]”).

### 4.11. Fractal Analysis

The following monofractal features were calculated: box fractal dimension averaged over 12 grid positions D_B_, SD for Dʙ, Dʙ_min_, Dʙ_max_, Dʙ with the highest r^2^, SE for Dʙ, Y-INT for Dʙ, mass fractal dimension averaged over 12 grid positions D_M_, SD for Dʍ, Dʍ_min_, Dʍ_max_, Dʍ with the highest r^2^, SE for Dʍ, Y-INT for FDʍ, D_X_ with the highest r^2^, SE for D_X_, Y-INT for D_X_, lacunarity Λ, lacunarity averaged over 12 grid positions Λ′, Λ_min_, Λ_max_, CV for Λ₍ɢ₎, and CV for Λ′₍ɢ₎. We used the box counting method for fractal analysis of greyscale images in FracLac plugin version 2016apr for ImageJ v1.52u as previously explained in full detail [36]. Grids consisted of boxes sized 5–575 pixels in linear 3-pixel increments. Grids were repositioned 12 within an image.

### 4.12. GLCM Analysis

Five GLCM features were calculated: angular second moment (ASM), inverse difference moment (IDM), contrast, correlation, and entropy using the GLCM Texture plugin for ImageJ v1.52u as previously explained in detail [37].

### 4.13. Calculation of Skewness, Kurtosis, IntDen, RawIntDen and Area of the Grey Level Range

The first-order statistical features were calculated in ImageJ using the run (“Measure”) function between the lower and upper grey level thresholds. For instance, analysis between grey levels of 160–180 was made using the Threshold (160, 180) setting command at Image > Adjust > Threshold and adjustment to “limit to threshold” in Analyze < Set Measurements < Limit to threshold.

### 4.14. Prognostic Evaluation

Values of the abovementioned features were averaged for the images available for each patient, followed by the prognostic evaluation using the ROC analysis with metastasis occurrence as the endpoint event. These tests compared the prognosticated and actual metastasis outcomes. The area under the rate of change curve (AUC) is a quantitative method commonly used to assess efficiency of discrimination with a binary endpoint. Discrimination is the capability of prognostic features to stratify patients with and without the actual metastasis occurrence. The AUC was calculated using continuous feature values. AUC values in the 0.0–0.5 range indicate an association with low risk and with high metastasis risk in the 0.5–1.0 range. AUC values farther away from its random performance midpoint at 0.5 indicate an improved discrimination efficiency. The data were categorized by dividing patients into low- and high-risk subgroups with an optimal cutoff selected using the X-tile 3.6.1 software (Yale University, New Haven, CT). The independence of each categorized prognostic classifier was tested using the Spearman’s correlation analysis.

### 4.15. Validation

The over-optimism of the ROC analysis (Stata/MP 13) analysis was corrected by the bootstrap internal validation with 1000 data resamples [38].

## 5. Conclusions

Our findings show for the first time the detailed distribution of the different intensities of pan cytokeratin immunostaining in breast tumors. On the one hand, the moderate and weak intensity immunostaining was the most prevalent and provided all of the prognostic value. On the other hand, the high intensity immunostaining represented only 15% of the specific staining and did not offer any prognostic value. Our computational analysis was not an attempt at the automation of the visual microscopy, but rather a discovery tool which revealed the intratumoral heterogeneity of discrete pan cytokeratin immunostaining intensities, their relative amount, spatial distribution, and prognostic value. Pan cytokeratin immunostaining requires even further detailed investigation to identify its most valuable prognostic clues and by that improve the prognostic performance that is currently achievable by analysis of the growth patterns of tumor malignant cells.

## Figures and Tables

**Table 1:** Prognostic evaluation of the pan cytokeratin immunostaining intensities.

Classification	AUC ^a^/*p*–Value									
	95% CI									
				Grey Level Ranges					Specific	Non-Spec
	0–255	0–130	130–160	160–180	180–200	200–220	220–240	240–255	0–220	220–255
GLCM features										
ASM	0.77/0.000 *	0.61/0.14	0.66/0.03 *	0.68/0.01 *	0.64/0.06	0.52/0.70	0.36/0.04 *	0.45/0.53	0.68/0.01 *	0.36/0.04 *
	0.66–0.87	0.44–0.70	0.56–0.81	0.56–0.81	0.50–0.77	0.38–0.67	0.22–0.46	0.38–0.59	0.57–0.78	0.24–0.47
Contrast	0.39/0.13	0.38/0.08	0.35/0.04 *	0.31/0.01 *	0.34/0.03 *	0.42/0.28	0.45/0.47	0.54/0.55	0.41/0.22	0.42/0.28
	0.28–0.53	0.24–0.54	0.19–0.43	0.19–0.43	0.22–0.48	0.29–0.56	0.33–0.58	0.42–0.68	0.30–0.53	0.30–0.55
Correlation	0.60/0.17	0.61/0.14	0.66/0.02 *	0.71/0.004 *	0.71/0.004 *	0.64/0.06	0.56/0.43	0.70/0.007 *	0.59/0.21	0.56/0.37
	0.43–0.75	0.43–0.74	0.60–0.82	0.60–0.82	0.58–0.83	0.52–0.76	0.43–0.69	0.58–0.81	0.44–0.74	0.43–0.70
IDM	0.75/0.000 *	0.61/0.14	0.66/0.03 *	0.68/0.01 *	0.64/0.06	0.53/0.70	0.41/0.20	0.48/0.75	0.68/0.01 *	0.41/0.21
	0.64–0.85	0.44–0.74	0.56–0.81	0.56–0.81	0.50–0.77	0.39–0.67	0.27–0.51	0.34–0.61	0.57–0.79	0.29–0.53
Entropy	0.28/0.002 *	0.40/0.17	0.34/0.03 *	0.32/0.01 *	0.37/0.06	0.48/0.81	0.65/0.04 *	0.54/0.59	0.32/0.01 *	0.61/0.12
	0.19–0.40	0.27–0.57	0.20–0.45	0.20–0.45	0.34–0.50	0.34–0.63	0.56–0.79	0.41–0.67	0.21–0.44	0.49–0.73
Fractal features										
D_B_	0.37/0.07	0.38/0.09	0.35/0.04 *	0.32/0.01 *	0.31/0.008 *	0.35/0.04 *	0.53/0.65	0.56/0.37	0.38/0.08	0.49/0.90
	0.24–0.50	0.24–0.52	0.22–0.48	0.19–0.45	0.19–0.43	0.23–0.47	0.41–0.66	0.44–0.69	0.25–0.51	0.34–0.62
SE for D_B_	0.49/0.90	0.63/0.07	0.65/0.04 *	0.64/0.05 *	0.63/0.08	0.55/0.48	0.40/0.17	0.49/0.85	0.50/0.95	0.47/0.65
	0.33–0.65	0.47–0.74	0.53–0.77	0.54–0.79	0.49–0.76	0.42–0.68	0.27–0.54	0.36–0.62	0.34–0.66	0.33–0.61
Λ	0.70/0.006 *	0.63/0.06	0.67/0.02 *	0.69/0.008 *	0.69/0.008 *	0.65/0.03 *	0.43/0.32	0.45/0.45	0.69/0.007 *	0.46/0.57
	0.57–0.82	0.50–0.77	0.54–0.79	0.56–0.82	0.57–0.81	0.53–0.77	0.31–0.55	0.32–0.57	0.57–0.81	0.33–0.59
First-order statistics ^b^										
Area	–	0.40/0.16	0.31/0.006 *	0.31/0.007 *	0.40/0.17	0.52/0.77	0.69/0.009 *	0.61/0.13	0.33/0.02 *	0.68//0/01 *
		0.26–0.55	0.18–0.43	0.19–0.43	0.27–0.53	0.38–0.66	0.67–0.80	0.48–0.74	0.20–0.45	0.57–0.79
Mean	036/0.05 *	0.51/0.91	0.46/0.58	0.36/0.05 *	0.35/0.03 *	0.41/0.21	0.37/0.007 *	0.68/0.01 *	0.57/0.34	0.55/0.48
	0.23–0.49	0.27–0.66	0.32–0.62	0.23–0.50	0.22–0.47	0.27–0.54	0.24–0.50	0.56–0.80	0.40–0.73	0.42–0.69
Kurtosis	0.67/0.02 *	0.50/0.98	0.54/0.52	0.55/0.52	0.59/0.20	0.61/0.12	0.62/0.10	0.69/0.01 *	0.56/0.68	0.64/0.05 *
	0.55–0.78	0.35–0.64	0.31–0.61	0.38–0.69	0.45–0.72	0.48–0.74	0.49–0.75	0.57–0.81	0.37–0.79	0.51–0.77

^a^ AUC in the 0.0–0.5 range indicates an association with low metastasis risk and with high risk in the 0.5–1.0 range. AUC values between 0.3–0.4 and 0.6–0.7 are considered indicators of fair, 0.2–0.3 and 0.7–0.8—of good, 0.1–0.2 and 0.8–0.9—of excellent, and 0.0–0.1 and 0.9–1.0—of almost perfect discrimination performance. ^b^ All first-order statistical features were calculated strictly within the designated intensity ranges without taking into account the white pixels of the 255 grey level. * p ≤ 0.05.

**Table 2:** Distribution of prognostic value among the pan cytokeratin immunostaining intensities.

**Pixel Intensity Ranges**										
Analytical method	Original	0–130	130–160	160–180	180–200	200–220	220–240	240–254	0–220	220–255
**Average AUC values ^a^**										
**AUC**	0.66	0.60	0.64	0.67	0.65	0.57	0.62	0.59	0.62	0.58
**AUC improvement in the narrow pixel intensity ranges ^b^**										
GLCM	-	0	0.10	0.19	0.15	0	0	0.10	0	0
Fractal analysis	-	0	0.18	0.20	0.20	0.02	0	0	0	0
First-order statistics	-	0	0	0	0.01	0.0	0	0.06	0	0
sum	-	0	0.28	0.39	0.36	0.02	0	0.16	0	0

^a^ AUC values were averaged for all features within each intensity range. Average AUC could only be calculated if values in the 0–0.5 range were adjusted to the 0.5–1.0 range (for instance, 0.34 = 0.66). ^b^ Improvements of AUC values observed in the narrow intensity ranges in comparison to the original images. Summed for each intensity range.

**Table 3:** Evaluation of the prognostic independence for image analysis features ^a^.

Feature	*p*-Value ^a^	HR	95% CI
Tumor size	0.03	1.07	1.01–1.15
0–255 mean	0.003	0.85	0.76–0.95
0–255 entropy	0.03	35.0	1.29–944
160–180 D_B_	0.04	0.00	0.00–0.39
240–255 kurtosis	0.002	1.02	1.01–1.03

^a^ Multivariate binary logistic stepwise regression analysis was performed by inclusion of the clinicopathological and image analysis features to capture the prognostic redundancy. The entry criterion was *p* ≤ 0.05 and the remain criterion was *p* ≤ 0.05. Abbreviations: HR = hazard ratio, CI = confidence interval, D_B_ = box-counting fractal dimension.

## Abbreviations

ASM	Angular Second Moment
IDM	Inverse Difference Moment
IntDen	Integrated Density, the product of Area and Mean Gray Value
RawIntDen	The sum of the pixel values in the image
GLCM	Gray-Level Co-Occurrence Matrix
ER	Estrogen receptor
PR	Progesterone receptor
Y-INT	Intersection with Y-axis
D_B_	Box fractal dimension
Λ	Lacunarity
AUC	Area under the ROC curve
ROC	Receiver operating characteristic
TNM	Staging system based on tumor size, lymph node spread, and metastasis.
